# Spray-coated electret materials with enhanced stability in a harsh environment for an MEMS energy harvesting device

**DOI:** 10.1038/s41378-021-00239-0

**Published:** 2021-02-09

**Authors:** Anxin Luo, Yixin Xu, Yulong Zhang, Mi Zhang, Xiaoqing Zhang, Yan Lu, Fei Wang

**Affiliations:** 1 School of Microelectronics, Southern University of Science and Technology, 518055 Shenzhen, China; 2 State Key Laboratory of AMS-VLSI, Institute of Microelectronics, University of Macau (UM), 999078 Macao, China; 3 Department of Electrical and Computer Engineering, FST, UM, 999078 Macao, China; 4 Engineering Research Center of Integrated Circuits for Next-Generation Communications, Ministry of Education, Southern University of Science and Technology, 518055 Shenzhen, China; 5 Department of Mechanical Engineering, Boston University, Boston, MA USA; 6 College of New Materials and New Energies, Shenzhen Technology University, 518118 Shenzhen, China; 7 Shanghai Key Laboratory of Special Artificial School of Physics Science and Engineering, Tongji University, 200092 Shanghai, China

**Keywords:** Physics, Electrical and electronic engineering

## Abstract

The charge stability of electret materials can directly affect the performance of electret-based devices such as electrostatic energy harvesters. In this paper, a spray-coating method is developed to deposit an electret layer with enhanced charge stability. The long-term stability of a spray-coated electret is investigated for 500 days and shows more stable performance than a spin-coated layer. A second-order linear model that includes both the surface charge and space charge is proposed to analyze the charge decay process of electrets in harsh environments at a high temperature (120 °C) and high humidity (99% RH); this model provides better accuracy than the traditional deep-trap model. To further verify the stability of the spray-coated electret, an electrostatic energy harvester is designed and fabricated with MEMS (micro-electromechanical systems) technology. The electret material can work as both the bonding interface and electret layer during fabrication. A maximum output power of 11.72 μW is harvested from a vibrating source at an acceleration of 28.5 m/s^2^. When the energy harvester with the spray-coated electret is exposed to a harsh environment (100 °C and 98% RH), an adequate amount of power can still be harvested even after 34 h and 48 h, respectively.

## Introduction

Electret materials have been used in various fields, such as pressure sensors, barometers and acoustic transducers in microphones^1–5^, thanks to the quasipermanent electric charge feature in electrets. Recently, electrets have been explored for use in new applications in MEMS vibration energy harvesters that are based on electrostatic induction^6–12^. A precharged electret can provide an electrostatic field between the static electrode and movable electrode for a long period of time. Driven by external vibration sources, the capacitance between the two electrodes is changed by varying either the overlapping area for the in-plane scheme^13^ or the gap distance for the out-of-plane closing scheme^6,14^. Therefore, charge flows between the electrodes and can be collected as harvested energy to provide sustainable power for wireless sensors with low power consumption. Compared with other types of energy harvesting principles, such as piezoelectric^15–20^, triboelectric^21–24^, and electromagnetic^25–29^ methods, electrostatic energy harvesters have been increasingly studied for their good compatibility with MEMS technology and integrated circuit (IC) fabrication processes^8,30–35^, which reduces the size and cost of devices and improves device reliability.

Kuehne et al.^36^ proposed a MEMS electrostatic energy harvester based on an out-of-plane gap closing scheme that was able to provide an output power of 4.28 μW with an external vibration at a frequency of 1 kHz and an amplitude of 0.2 g. Suzuki et al.^13^ reported a MEMS in-plane electret generator for energy harvesting applications. A total output power of 1 μW could be obtained at an external excitation of 2 g and 63 Hz. Nakano et al.^37^ developed a MEMS rotational electret energy harvester for capturing the kinetic energy of human motion and could harvest 3.6 μW power with a rotation of 1 rps.

The performance of an electrostatic energy harvester is highly dependent on the precharged electret material. Generally, the harvested power from vibration is proportional to the square of the charge density in electret materials^10^, which might decay once exposed to harsh environments at high humidity levels or high temperatures. Hence, it is necessary to promote the charge stability of electret materials for improving the performance of electrostatic energy harvesters. Thermal treatment during electret charging is generally performed to improve charge uniformity and stability^38,39^. In addition, Chen et al.^40^ proposed a charging method based on interfacial polarization in double-layer media that could achieve both excellent charge stability and high charge density. Thyssen et al.^41^ also improved the charge stability at high temperature and high humidity by controlling the crystallinity of polypropylene electret material; this improvement was achieved by mixing isotactic-polypropylene and atactic-polypropylene. In addition to these endeavors, some surface treatment methods and a spray-coating method were proposed and proved to be good methods for film deposition and enhanced performance according to our previous work^20,42–44^.

In this work, we develop a spray-coating method for the deposition of electret materials with nanoparticles. The charging and decay processes of electret layers have been investigated under harsh conditions of high humidity and high temperature. The long-term stability of the prepared electrets are evaluated for more than 500 days, and a second-order linear model is proposed to interpret the experimental data with better accuracy than the conventional deep-trap model. Furthermore, to verify the application of spray-coated electret materials, an electrostatic energy harvester with an out-of-the-plane gap closing scheme is fabricated using advanced MEMS technology. The prepared MEMS energy harvester can successfully harvest energy from random vibrations with stable performance at high temperature and high humidity.

## Results and discussion

### Surface morphology and charge stability over 500 days

**Fig. 1:**
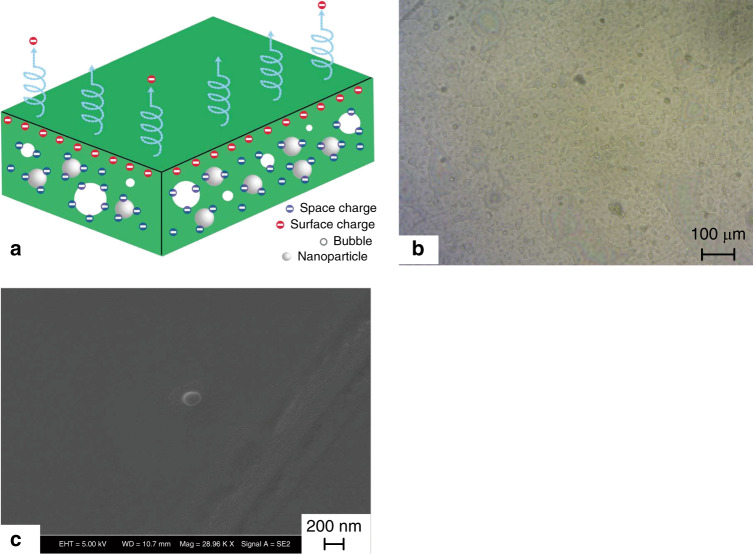
The proposed spray-coating method for electret material with nanoparticles. **a** Schematic of the electret material deposited by spray coating and small bubbles and PS nanoparticles are introduced by spray coating, which could be helpful for trapping more space charges; **b** photograph of a spray-coated electret with many bubbles; **c** SEM image of a nanoparticle on an electret layer; Table 1: Samples prepared by spin and spray-coating methods

**Fig. 2:**
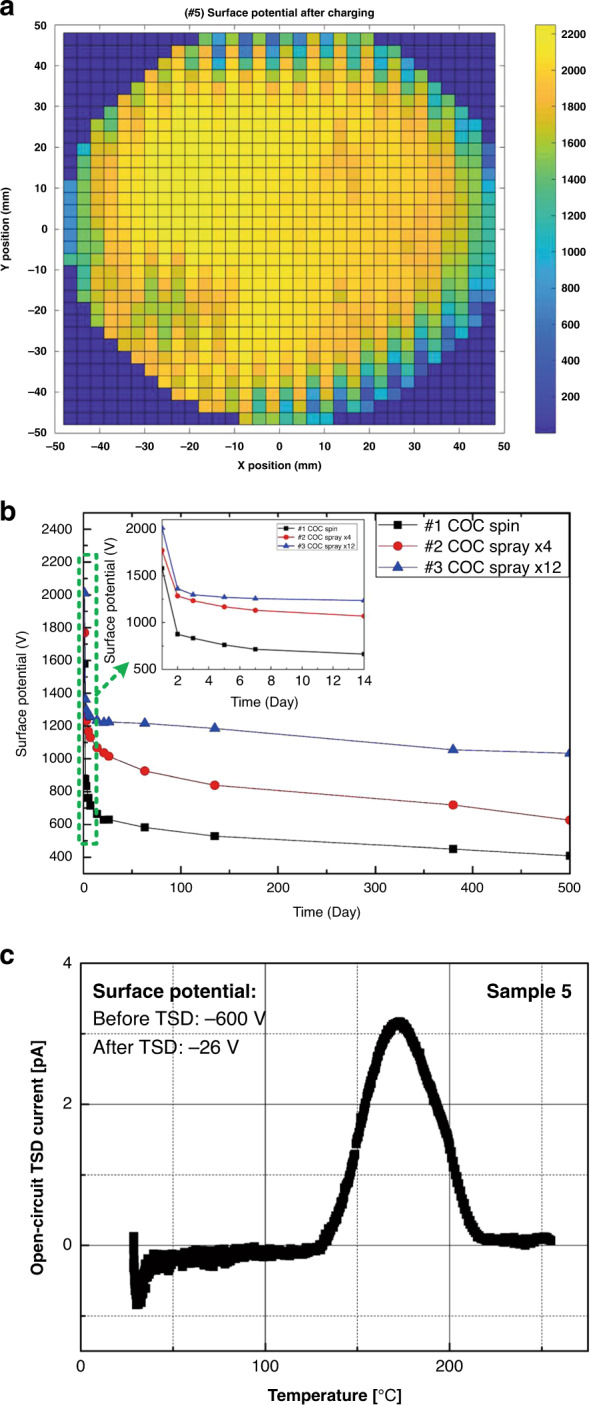
Experimental results of spray-coated electret with nanoparticles (Sample 5). **a** Surface potential distribution of Sample 5 after corona charging, **b** surface potential decay over 500 days, and **c** open-circuit TSD current spectra of Sample 5

Spray coating has been proven to be a useful method to enhance the stability of electrets. By adding polystyrene (PS) nanoparticles to the electret, the performance of the cyclic olefin copolymer (COC) electret can be further enhanced. During spray coating, microbubbles with nanoparticles are introduced to the electret layer, as shown in Fig. 1a. The spray coater and spray-coating process have been described in our previous report^44^ and in the Supplementary Materials (Fig. S1). The microbubbles and nanoparticles are capable of retaining the space charge; therefore, they help prevent charge decay. Microscopic photographs of the bubbles and scanning electron microscope (SEM) images of the particles in the spray-coated electret layer are shown in Fig. 1b, c, respectively. To further explore the effect of coating methods on charge stability, the five electret samples listed in Table 1 are prepared with different concentrations by spin and spray-coating methods. The five electret materials were charged with a typical corona charging setup^38^. A detailed description of the surface potential measurement method is introduced in the Supplementary Materials. Figure 2a shows the uniform distribution of the surface potential for Sample 5 after corona charging.

**Table 1:** Samples prepared by spin and spray coating

No.	COC Conc. (g/100 mL)	Nanoparticles conc. (μL)	Deposition method	Parameter
1	20	0	Spin	500 rpm
2	1	0	Spray	4 cycles
3	1	0	Spray	12 cycles
4	1	PS, 200	Spray	12 cycles
5	1	PS, 400	Spray	12 cycles

To evaluate the charge stability of the coated electrets over a long-term period, the samples were kept in a plastic box at room temperature (25 °C) and normal humidity (30–50% RH) for 500 days. As shown in the inset plot of Fig. 2b, all three samples exhibit a quick decrease in surface potential during the first 3 days, which gradually stabilizes afterward. After 14 days, the surface potential of Sample 1 decreases from 1600 to 700 V, while approximately 400 V remains after 500 days; this value is only ~25% of the initial value. In contrast, for the spray-coated COC sample (#3), more than 1100 V of the surface potential is retained after the long-term test. Considering the effective thickness of this sample is 20 µm, a high surface charge density of 1.15 mC/m^2^ is maintained after 500 days, which proves the excellent stability of the space charge. The thermal stability of charge trapping in electrets can also be verified from thermal stimulated discharge (TSD) measurements, as demonstrated in Fig. 2c. The backside of Sample 5 was metalized and the open-circuit TSD current was measured. The measured current exhibits a peak at ~180 °C, indicating that the electret samples can maintain good stability at room temperature.

### Second-order linear model for charge decay in harsh environments

**Fig. 3:**
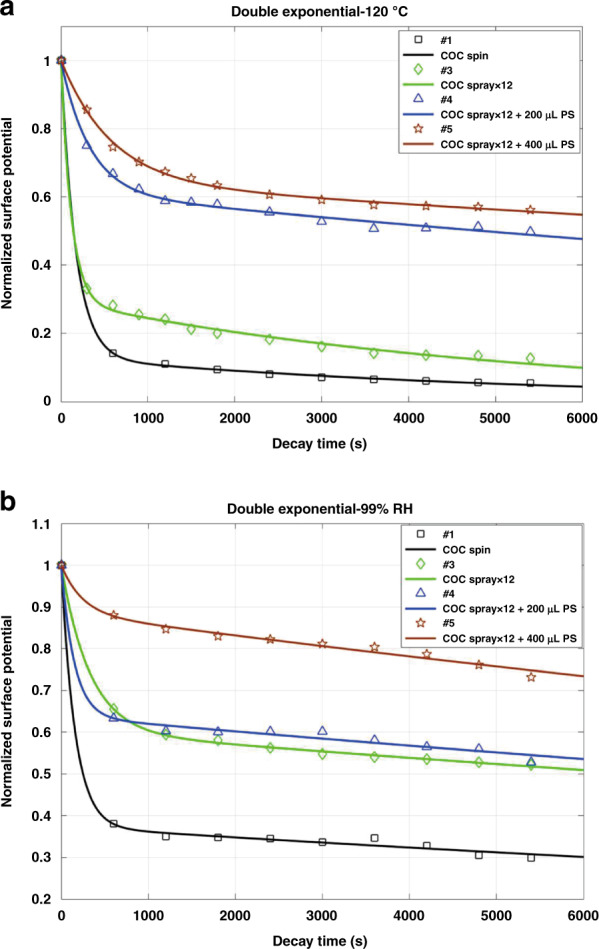
Charge decay results fitted by a second-order linear model. **a** Fitting result of the charge decay in electrets exposed to high temperature (120 °C), **b** Fitting result of the charge decay in electrets exposed to high humidity (99% RH). The fitting parameters of the charge decay process at high temperature (120 °C) and high humidity (99% RH) are listed in Tables 2 and 3, respectively

To further explore the charge decay mechanism of the spray-coated electrets in harsh environments, we tested the samples under high temperature (120 °C) and high humidity (99% RH) conditions for 1.5 h according to the literature^45–48^. The experimental results are shown in Fig. 3a, b (marks represent the measured data). A second-order linear model is proposed to analyze the decay of the surface potential and fits better to the experimental results than the traditional deep-trap model.

The charge decay process was first analyzed with the traditional deep-trap model, as shown in the Supplementary Materials and Fig. S3. From the fitting result, we can see that the deep-trap model is able to fit the experimental result with a coefficient of determination *R*^*2*^ ranging from 0.91 to 0.99, which presents the degree of fitting. Despite this fitting result, we cannot observe a clear relationship between the performance of the electret and the carrier-transit time or relaxation time. From the fitting, qualitative analysis shows that the electret with good charge stability (#5) in the humidity test and temperature test has the longest carrier-transit time and relaxation time. Therefore, the deep-trap model explains the mechanism of charge decay in the electret only to some degree but fails to interpret the more detailed aspects of the charge decay mechanism.

In this paper, we propose a second-order linear model for the charge decay of electrets, which has seldom been reported^49^. Unlike the deep-trap model, the second-order linear model considers both the surface charge and space charge. This model describes the charge decay process by the following equation with two exponential parts:

1 $$\frac{{V(t)}}{{V(0)}} = a_1e^{ - b_1t} + a_2e^{ - b_2t}$$

where *a*_1_ and *a*_2_ are exponential coefficients and *b*_1_ and *b*_2_ are time coefficients. This model describes the charge decay process with respect to the two different types of charges inside the electret. The space charge decays due to internal factors, including ohmic conduction, charge drift and diffusion, while the surface charge decays by ion deposition from the environment outside the electret. The overall charge decay in the COC electrets can be explained by the quick decay of the surface charge in addition to the relatively slow decay of the space charge. Both of these charges can be described with an exponential function.

We apply the second-order linear model to fit the experimental data. Figure 3a, b show the experimental results (the marks) and fitting curves. The calculated exponential coefficients *a*_1_ and *a*_2_ and time coefficients *b*_1_ and *b*_2_ of the fitting curves are listed in Tables 2 and 3 for the stability tests performed at high temperature (120 °C) and high humidity (99% RH), respectively. From the results, it is confirmed that the decay data perfectly fits with the curve, and excellent *R*^*2*^ values ranging from 0.9868 to 0.9999 are achieved. The second-order linear model fits the experimental results with much better accuracy than the conventional deep-trap model.

**Table 2:** Fitting parameters of the charge decay process at high temperature (120 °C)

	#1 COC Spin	#3 COC Spray × 12	#4 COC spray × 12 + 200 μL PS	#5 COC spray × 12 + 400 μL PS
*a*_1_	0.8704	0.7071	0.3848	0.3557
*b*_1_	−5.9 × 10^−3^	−8.4 × 10^−3^	−3.0 × 10^−3^	−1.7 × 10^−3^
*a*_2_	0.1296	0.2927	0.6130	0.6444
*b*_2_	−1.8 × 10^−4^	−1.81 × 10^−4^	−4.19 × 10^−5^	−2.71 × 10^−5^
*R*^2^	0.9999	0.9981	0.9950	0.9975

**Table 3:** Fitting parameters of the charge decay process at high humidity (99% RH)

	#1 COC Spin	#3 COC Spray × 12	#4 COC Spray × 12 + 200 μL PS	#5 COC Spray × 12 + 400 μL PS
*a*_1_	0.6259	0.3974	0.3619	0.1149
*b*_1_	−6.3 × 10^−3^	−3.1 × 10^−3^	−6.7 × 10^−3^	−4.2 × 10^−3^
*a*_2_	0.3741	0.6025	0.6381	0.8851
*b*_2_	−3.62 × 10^−5^	−2.81 × 10^−5^	−2.92 × 10^−5^	−3.12 × 10^−5^
*R*^2^	0.9983	0.9994	0.9946	0.9868

Based on the fitting data, we can clearly see that the space charge plays a more dominant role in all three spray-coated electrets (larger *a*_2_) compared with the spin-coated sample, which is mainly due to microbubbles. Furthermore, *a*_2_ increases with the amount of PS nanoparticles because an increase in the number of internal defects can enrich the space charge. For both tests performed at high temperature and high humidity, the slowest charge decay is observed for the spray-coated electret with PS nanoparticles (Sample 5) thanks to the large amount of space charge. It should also be noted that for all the samples, a higher value of *a*_1_ is obtained from the thermal test (Table 2) compared with that value from the humidity test (Table 3). This result is because the high temperature decays both the surface charge and space charge, while the humid environment mainly attacks the surface charge. This second-order linear model can confirm that the defects introduced by the spray-coating method with nanoparticles traps a good amount of space charge, which will enhance the charge stability of the coated polymer electret.

### Energy harvesting device with a spray-coated electret and MEMS technology

**Fig. 4:**
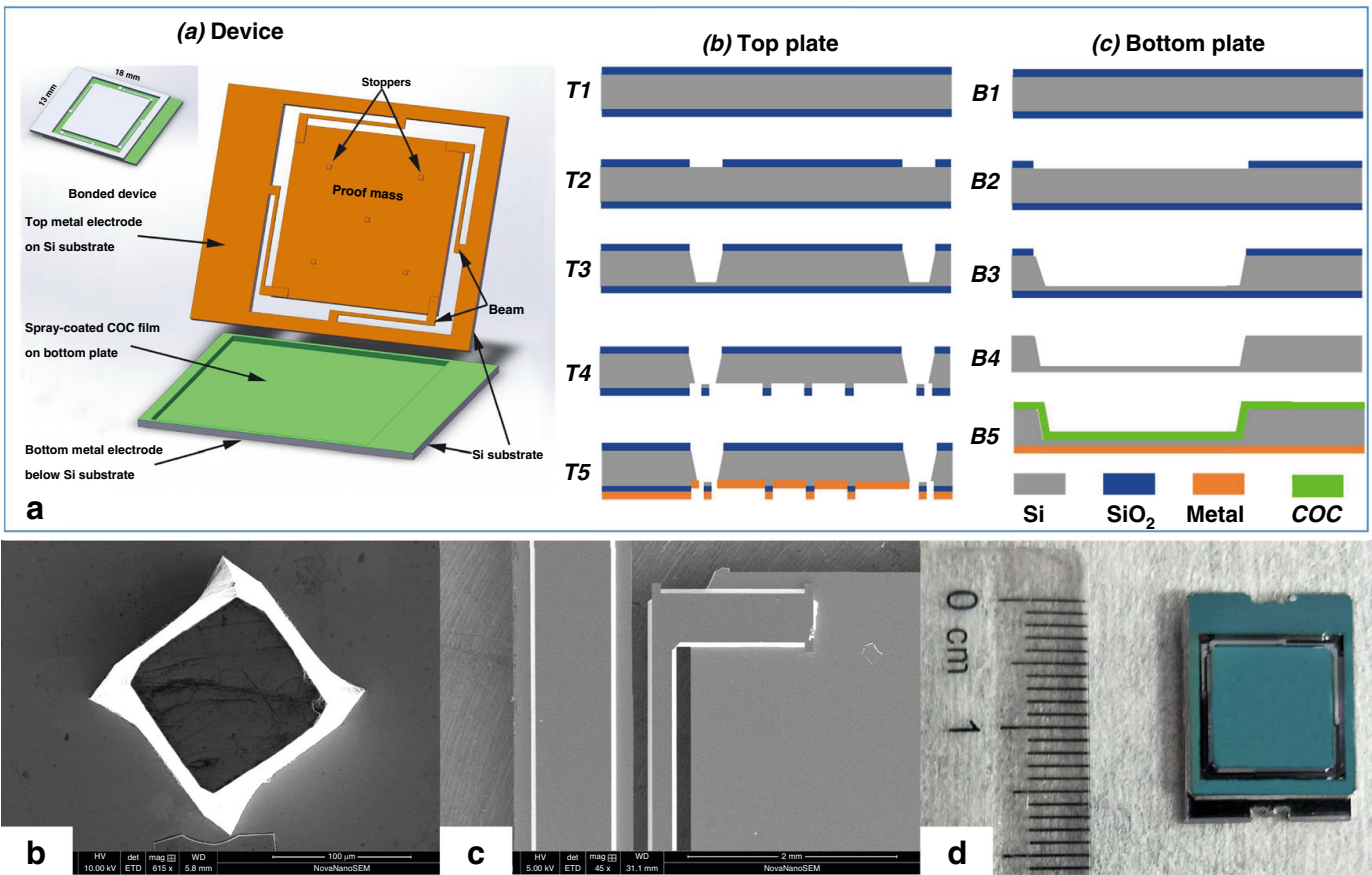
Fabrication process of the energy harvester device and its photos. **a** Schematic and fabrication process of the electrostatic energy harvester device, which is composed of a top plate and bottom plate with MEMS technology, SEM images of the **b** stopper and **c** beam on the energy harvester device with corner compensation, and **d** electrostatic energy harvester after low-temperature bonding. The size of this whole device is 13 × 18 mm^2^

To verify the enhanced stability, we apply a spray-coated electret in electrostatic MEMS energy harvesters and evaluate the harvested power in harsh environments. Figure 4a shows an electrostatic energy harvester that is designed and fabricated with spray-coated COC and PS nanoparticles using advanced MEMS technology. The device mainly consists of two parts, namely, the top plate and bottom plate. The top plate is designed with a proof mass suspended by four beams, which will vibrate when driven by ambient vibration. Five stoppers are designed on the proof mass to prevent the “pull-in” effect during vibration^50^. The bottom plate is mainly constructed with a cavity, which limits the maximum amplitude of the device. An electret layer is uniformly coated on the bottom plate by spray coating to generate an electrical field. On both plates, a metal layer is deposited as the electrode to lead out the harvested power.

As Fig. 4a shows, two parts of the device are fabricated individually based on 4-inch wafer MEMS technology. The detailed fabrication process is outlined below.

For the top plate, T1: First, thermal oxidation is performed to grow a 2-μm oxide layer on both sides of the silicon wafer.

T2: Lithography is used to pattern the top-side SiO_2_ layer, while the bottom side of the wafer is protected by the photoresist. The exposed SiO_2_ is etched by a buffered oxide etchant (BOE).

T3: With the SiO_2_ mask, KOH solution is used as an etchant to form the proof mass and beams. Corner compensation should be carefully considered for the mask design to maintain the shape of the mass during wet etching.

T4: Afterward, dry etching is applied from the bottom side of the wafer to release the four beams and stoppers. The wafer is patterned by lithography.

T5: Finally, a metal layer of Al/Cr (100 nm/15 nm) is sputtered as the electrode, and the top plate is fabricated.

For the bottom plate, the fabrication process is much simpler than that for the top plate.

B1-B2: Similar to T1 and T2, SiO_2_ is thermally grown and then patterned with photolithography followed by BOE etching.

B3: With SiO_2_ as the mask layer, a cavity with a depth of 300 μm is formed by KOH wet etching.

B4: The entire SiO_2_ layer left is stripped by BOE.

B5: The Al/Cr (100 nm/15 nm) metal layer is sputtered as the bottom electrode. A 90-μm COC electret layer is spray coated.

Then, the bottom plate is ready for corona charging. The voltage of the mesh grid is set at −800 V, and the overall charging time is 5 min. The surface potential of the sprayed COC electret is stabilized to −550 V 30 min after charging.

The two plates are bonded together at low temperature to avoid severe charge decay of the electret material. The spray-coated electret material acts both as the electret layer and adhesive layer during the bonding process. A bonding pressure of 0.01 MPa and a temperature of 100 °C are applied for 10 min to achieve reliable bonding strength. According to the thermal test above, the electret layer can survive the bonding process without a severe loss in charge density. Figure 4b, c illustrate the SEM images of the stopper and beam structures on the energy harvester device. Finally, Fig. 4d demonstrates the image of the fabricated energy harvesting device after bonding, with a compact size of 13 × 18 mm^2^.

**Fig. 5:**
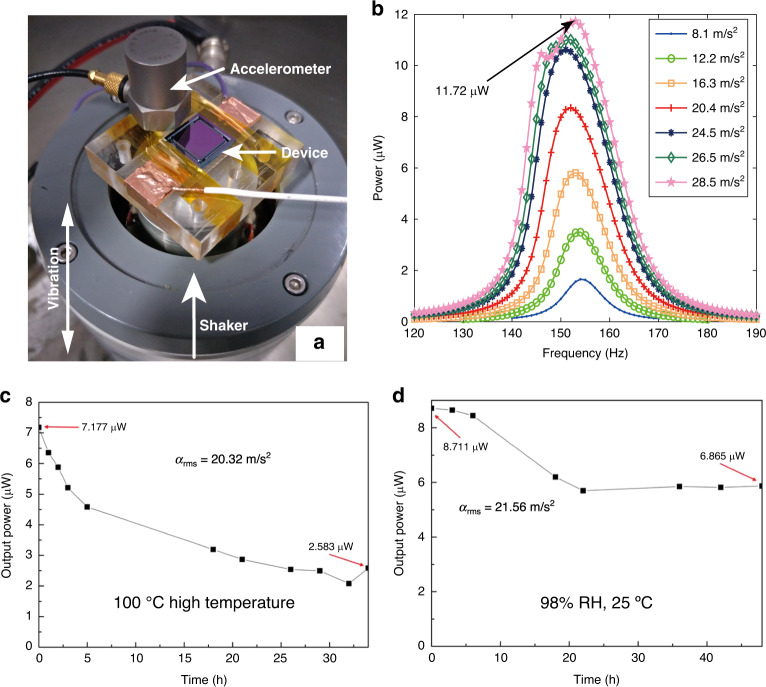
Characterization of the device. **a** The device is characterized by using a shaker with an accelerometer; **b** The frequency response of RMS powers vs. frequencies at different accelerations; The maximum RMS output power of the device after being placed **c** on 100 °C hotplate and **d** in 98% RH, 25 °C humidity cabinet for different time

The performance of the spray-coated electret-based electrostatic energy harvester device is characterized using a shaker to mimic the ambient vibration source, as shown in Fig. 5a. An accelerometer is fixed together with the energy harvester to monitor the real-time acceleration.

With a series test circuit^51,52^, the optimal load resistance of the energy harvester is measured as 21 MΩ, as shown in the Supplementary Information (Fig. S4); this value is used for all the following tests unless otherwise noted. Figure 5b shows the frequency response of the device when the vibration frequency is tuned from 140 to 190 Hz. Root mean square (RMS) acceleration ranging from 8.1 to 28.5 m/s^2^ is applied, and the amplitude of the device at different accelerations can be found in the Supplementary Information (Fig. S5). A maximum RMS output power of 11.72 μW is harvested at a resonant frequency of 154 Hz with a quality factor of approximately 10. The electrostatic energy harvester with a spray-coated electret shows excellent performance compared with previously published devices. A plateau can be seen in the RMS power output at an RMS acceleration above 26.5 m/s^2^, where collision begins to occur^50^. The output power of the device driven by random vibration signals is also tested, as depicted in the Supplementary Information (Fig. S6).

The enhanced charge stability is the major advantage of the spray-coated electret in this work. Therefore, the MEMS energy harvester is finally tested in a harsh environment at 100 °C and 98% RH. As shown in Fig. 5c, the electrostatic harvester withstands a significant decrease in the RMS output power after the first 5 h. However, it can still maintain an RMS output power of 2.5 μW after enduring long-term baking for 34 h. This result shows that there is a good amount of space charge in the sprayed electret that can survive at high temperatures. Similar phenomena have also been observed for the CYTOP layer enhanced by nanoclusters^53^. For the device at high humidity, as shown in Fig. 5d, a slight decrease in the RMS output power is noticed after 20 h, which is mainly due to the quick loss of the surface charge at high humidity. However, the device output stabilizes at 6.87 μW for the following 28 h, proving that the space charge in the electret layer can resist further influence from the high humidity environment.

## Conclusions

The charge decay of the electret over 500 days confirmed that the spray-coating method with nanoparticles could significantly improve the charge stability over a long-term period. A second-order linear model was proposed to analyze the decay of the surface potential, which fit the experimental results better than the traditional deep-trap model. The microbubbles and nanoparticles could trap more space charge inside the spray-coated electret, which was beneficial for enhancing the charge stability of electret materials in harsh environments. The spray-coated electret was successfully applied in an electrostatic energy harvester, which provided a maximum RMS output power of 11.72 μW. The device showed excellent stability for energy harvesting in harsh environments. Adequate power could be harvested after dozens of hours of exposure at high temperature or high humidity. This enhanced charge stability shows promise for the application of spray-coated electrets in energy harvesters, microphones, and other related devices.

## Materials and methods

### Coating and charging method for the electret material

All electrets were deposited on a 4-inch silicon wafer, and the electret material used in this work was a cyclic olefin copolymer (COC, TOPAS 8007S-04 pellet from Topas Advanced Polymers GmbH, Germany). First, 1 g or 20 g of COC pellets were dissolved in 100 mL of toluene at room temperature to achieve different electret solutions. The COC solution should be mixed for more than 12 h with a magnet stirrer before the total dissolution of COC particles. For the spin-coated electret, Sample 1, 200 g/L electret solution was used at a spinning speed of 500 rpm for 30 s. For the spray-coated electret samples, Samples 2–5, a solution at a low concentration of 10 g/L was applied for better mobility. An SC-6 spray coater (Suzhou MEMStools Semiconductor Technology, China) was employed for electret deposition. During spray coating, the wafer was baked at 65 °C to dry out the electret solution. Finally, all samples were baked on a 120 °C hotplate for 30 min to stabilize before corona charging.

We applied a high voltage of 7000 V for the corona tip, while a bias voltage of 2000 V was applied for the mesh grid during the charging process. For all samples, the corona charging process was performed at room temperature for 600 s. An electrostatic voltmeter (Trek, model 347, America) was used with a scanning probe stage system to measure the distribution of the charged surface potential.

### Fabrication and characterization of the energy harvesting device

Both the top plate and bottom plate of the energy harvester were fabricated based on a 4-inch (100) wafer with a thickness of 400 μm. A 40% KOH solution at 50 °C was used as the etchant for wet etching, and the etching depth was set to approximately 280 μm. During the dry etching process, AZ4620 (AZ Electronic Materials, Luxembourg) was used to form an ~12-μm photoresist layer. The wafer was dry etched by ICP for ~1500 s (O_2_: 44 sccm, SF_6_: 86 sccm, pressure: 0.0047 MPa). Finally, a 10 g/L COC solution with 400 μL of PS nanoparticles was spray coated for 12 cycles to form a 90-μm COC electret layer. During characterization, the device was mounted on a shaker. The shaker was driven by an excitation signal that was generated from a power amplifier (Brüel&Kjær, 2719, Denmark), a signal generator (Brüel&Kjær, LAN-XI 3160, Denmark) and a power amplifier (Brüel&Kjær, 2719, Denmark).

## Supplementary information

Supplementary materials

Supplementary materials with revision highlighted
