# Supplementary material for: Spray-coated electret materials with enhanced stability in a harsh environment for an MEMS energy harvesting device
Source: Microsyst Nanoeng. 2021 Feb 9;7:15. doi: 10.1038/s41378-021-00239-0 (PMC8433343; doi:10.1038/s41378-021-00239-0)
Supplement: Supplementary file 2 — Supplementary materials with revision highlighted [file 41378_2021_239_MOESM2_ESM.docx]

Supplementary Materials

**Spray Coated Electret Materials with Enhanced Stability in Harsh Environment for MEMS Energy Harvesting Device**

Anxin Luo^1,2,3,4,*^, Yixin Xu^5,*^, Yulong Zhang^6^, Mi Zhang^7^, Xiaoqing Zhang^7^, Yan Lu^2,3^ and Fei Wang^1,4,**^

^1^School of Microelectronics, Southern University of Science and Technology, Shenzhen 518055, China

^2^State Key Laboratory of AMS-VLSI, Institute of Microelectronics, University of Macau (UM), Macao 999078, China

^3^Department of Electrical and Computer Engineering, FST, UM, Macao 999078, China

^4^Engineering Research Center of Integrated Circuits for Next-Generation Communications, Ministry of Education，Southern University of Science and Technology, Shenzhen 518055, China

^5^Department of Mechanical Engineering, Boston University, Boston, MA, USA

^6^College of New Materials and New Energies, Shenzhen Technology University, Shenzhen 518118, China

^7^Shanghai Key Laboratory of Special Artificial School of Physics Science and Engineering, Tongji University, Shanghai 200092, China

* These authors contribute equally to this work

** Corresponding author: Tel.: +86 755 8801 8509. Fax: +86 755 8801 0000.

E-mail addresses: wangf@sustech.edu.cn

**Spray coating method**

For the spray coating method, we have prepared the electret solution at first. 1 g COC pellets were dissolved in 100 mL toluene at room temperature to achieve an electret solution with good mobility. The COC solution should be mixed for more than 12 hours with magnet stirrer before the total dissolution of COC particles. After that, nanoparticles were added and mixed into the solution with 4 hours ultrasonic stirring. The principle of spray coating process is demonstrated in Fig. S1a. Similar to the spray coating process for the photoresist, the mixed solution was stored in the pressure tank, and then a syringe pump is used to pump out the solution with a pump pressure of 0.2 MPa and spray the polymer on the surface of silicon wafer with the spray pressure of 0.1 MPa. During the coating process, we have used a SC-6 spray coater (Suzhou MEMStools Semiconductor Technology, China) as shown in Fig. S1b to deposit the COC electret layer. During coating, the wafer was baked at 65 ºC so that the polymer solution could dry out and the electret would stabilize on the silicon wafer. The spray nozzle scans across the wafer for each cycle and after each cycle the silicon wafer rotates by 180 degrees as shown in Fig. S1a, which provides better coating and more uniform COC layer. Samples with different thickness can be acquired simply by tuning the cycle numbers. The coated wafers were finally baked with hotplate at 180 ºC for 30 minutes for thermal curing and evaporating the toluene before the following corona charging.


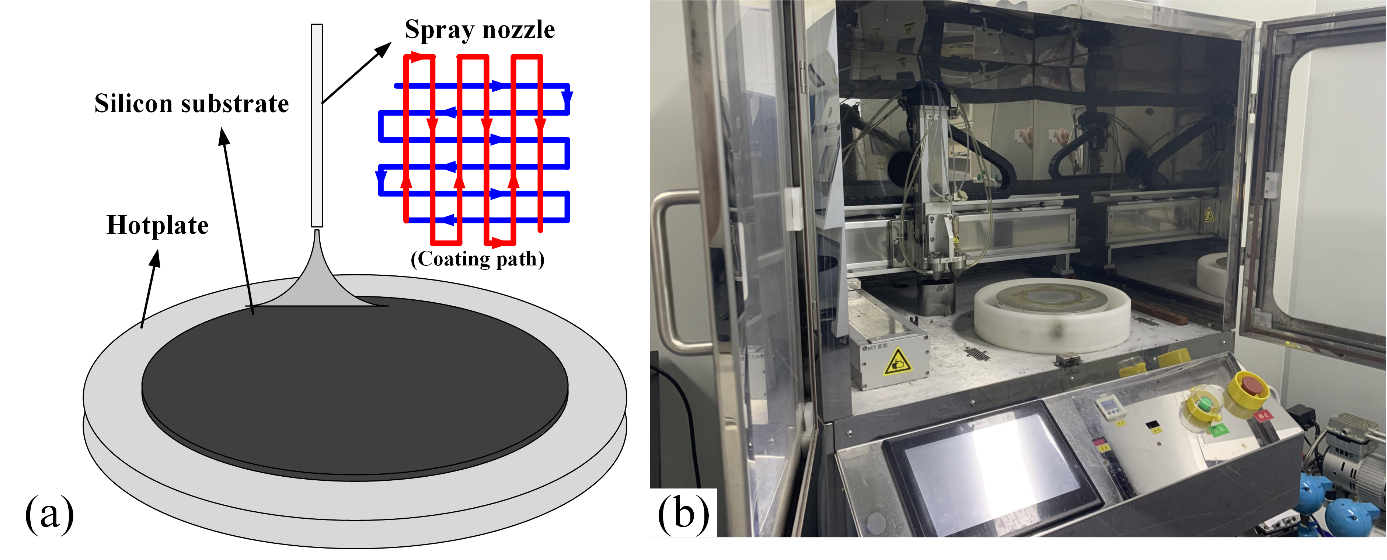


Figure. S1: (a) Principle of spray coating process; (b) Spray coater for the polymer electret coating.

**Surface potential measurement method**

The surface potential measurement system is composed by an electrostatic voltmeter (Trek, model 347) to detect the surface potential of electret sample in small area as shown in Fig. S2a and a probe stage to scan the sample step by step as demonstrated in Fig. S2b.

**
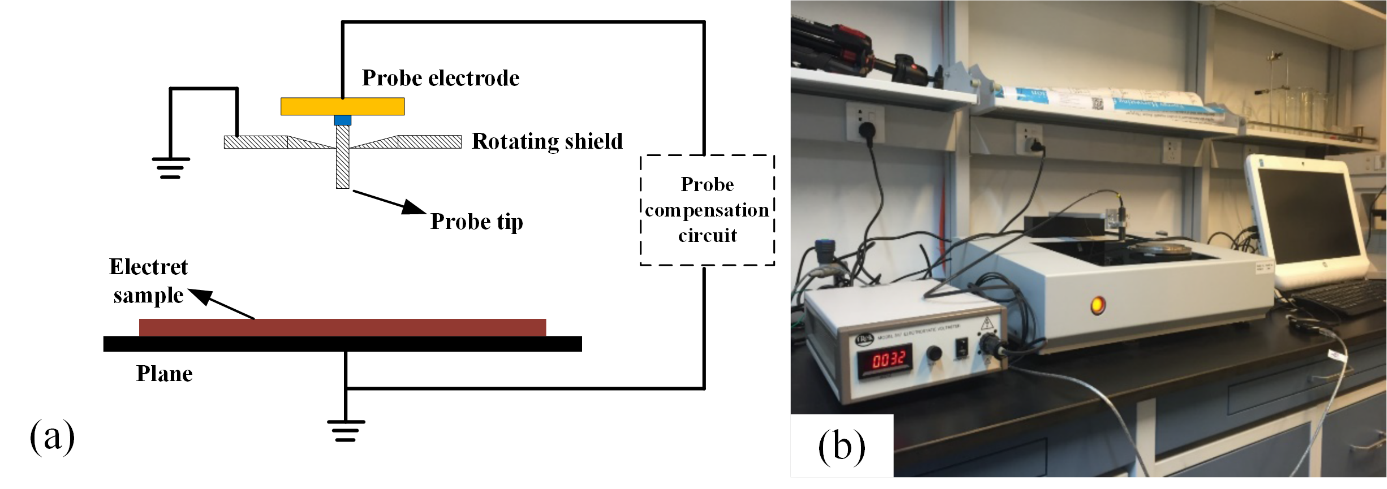
**

Figure. S2: (a) Principle of the surface potential measurement with distance-compensated electrostatic voltmeter for electret layer; (b) Image of the surface potential measurement system.

**Deep trap model**

Deep trap model is widely accepted and applied in the research of electret. It explains the decay of charges from internal elements: Ohmic conduction or drift and diffusion of real charges. In deep trap model, the substrate is considered as metallized electrode with finite ohmic conductivity and connected to the ground. The electret is considered as dielectric with zero ohmic conductivity. In such situation, diffusion plays a much minor role comparing to ohmic conduction and charge drift, thus, it is ignored. Considering open current conditions and Poisson’s equation, deep trap model concludes the decay trend by

 (1)

where *V(t)* is the surface potential at t seconds after charging and *V(0)* is the initial surface potential of electret sample. We defined

$t_{0\lambda}=s^{2}/\mu V\left(0\right)$ (2)

as carrier-transit time, which is the time an excess charge takes to transit from *x=0* to *x=s* and reaches the electrode.

$\tau'=\varepsilon_{0}\varepsilon_{r}/\mu_{0}eN_{t}$ (3)

is the relaxation time.$\varepsilon_{0}$ and $\mu_{0}$ are the physical constant called vacuum permittivity and vacuum permeability, respectively. $\varepsilon_{r}$ is a ratio relative to the vacuum permittivity, and *N_t_* is the trap concentration. The transit time $t_{\lambda}^{'}$equals to

$t_{\lambda}^{'}=\tau^{'}\ln\frac{\tau^{'}}{\tau^{'}-t_{0\lambda}}$ (4)

The deep trap model is applied to our experimental data. Fitting of deep trap model is done in the MATLAB. Tool box named curve fitting is used. The surface potential is first normalized by

$V_{N}\left(t\right)=V\left(t\right)/V\left(0\right)$ (5)

where *V_N_(t)* refers to the normalized surface potential at *t.* The fitting results of electret samples decayed in harsh environment by deep trap model are demonstrated in Fig. S3, where (a),(b) shows the fitting results of the electret decayed in high temperature (120 ºC) and (c),(d) illustrates the fitting results of the electret decayed in high humidity, respectively. Moreover, the coefficient of determination *R^2^* is also listed, which presents the degree of the fitting. Higher *R^2^* represents a better fitting.


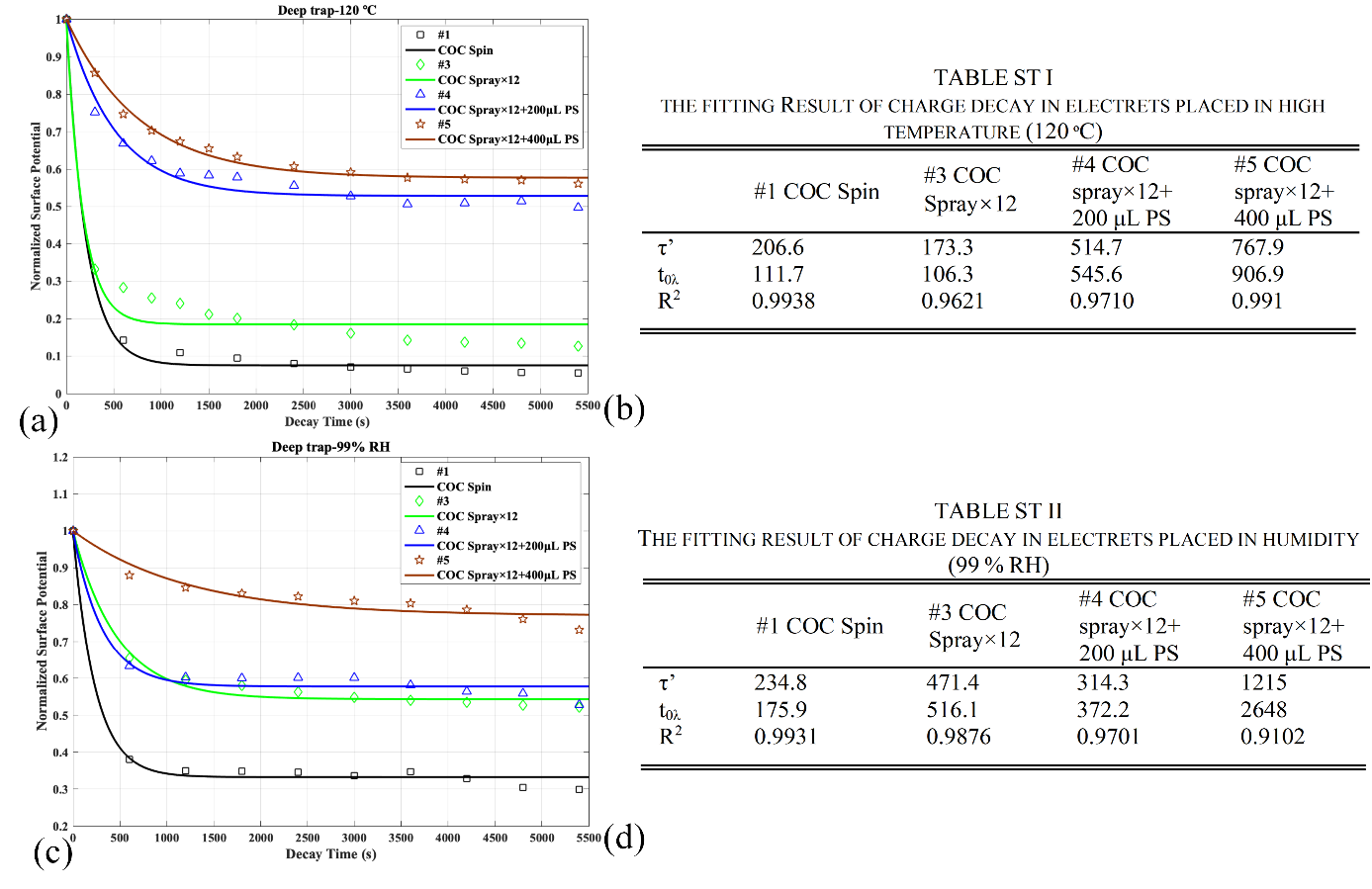


Figure. S3: The Fitting Result of charge decay in electrets placed in (a),(b) high temperature (120 ºC) and (c),(d) high humidity (99 % RH) by deep trap model.

**Characterization of the electret based energy harvester**

During the test, we have tested the output voltage and RMS output power excited by the acceleration of 0.8g vibration with different load resistance as figure. S4 below demonstrates. The optimal load resistance of this device is 21 MΩ, where the device can output the maximum power, and the output voltage is 12.8 V (peak to peak) under this circumstance. When the load resistance is 31 MΩ, the output voltage at this time is 15 V (peak to peak) which is close to the open-circuit voltage.

**
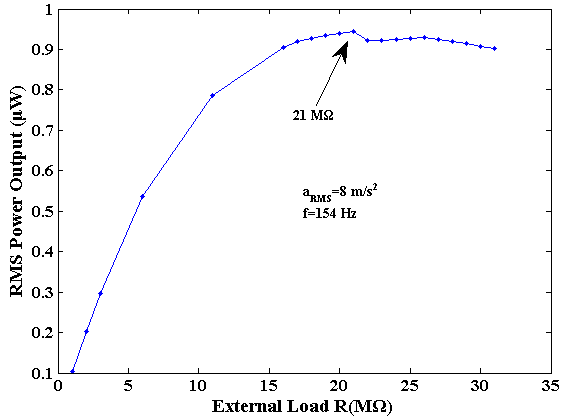
**

Figure. S4: The output power of the device with different external load resistance at the RMS acceleration of 8 m/s^2^.


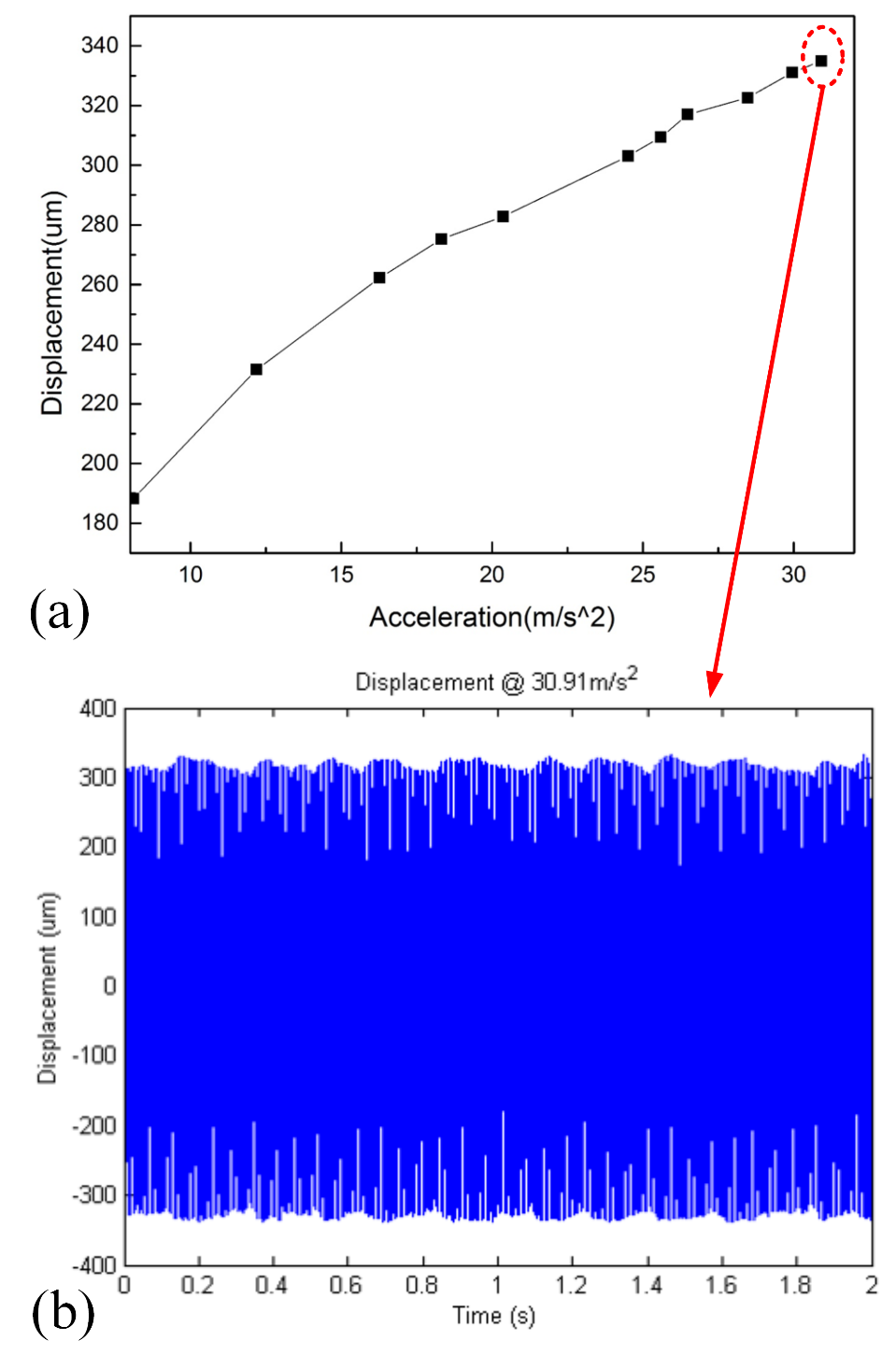


Figure. S5: (a) The amplitude of the device under different vibration acceleration; (b) The amplitude fluctuates violently at the RMS acceleration of 30.91m/s^2^.


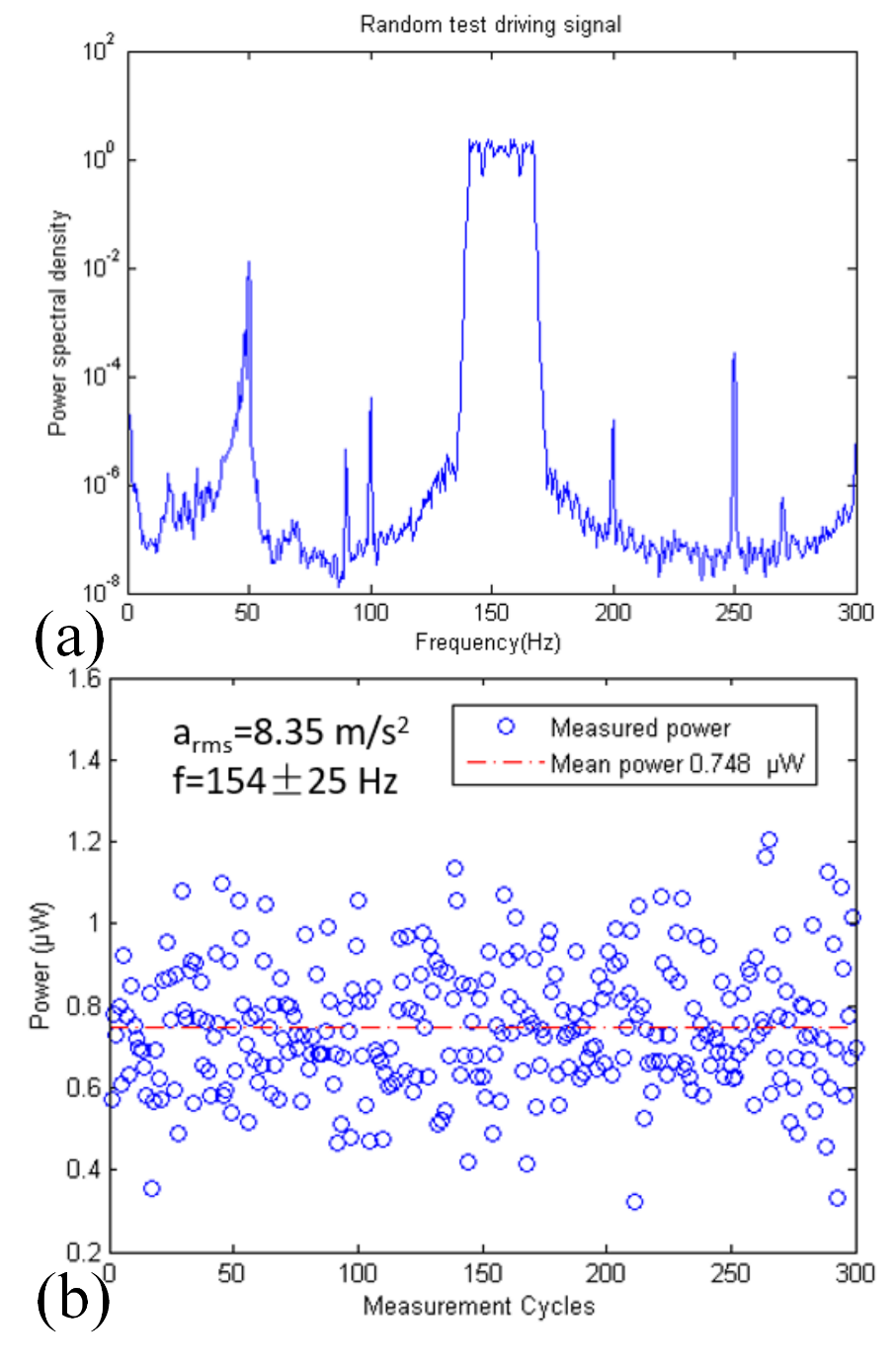


Figure. S6: (a) The driving frequency in random test is 154 ± 25 Hz (a) The RMS output power during random vibration test with an average RMS acceleration of 8.35 m/s^2^.
